# Hypochondroplasia, Acanthosis Nigricans, and Insulin Resistance in a Child with FGFR3 Mutation: Is It Just an Association?

**DOI:** 10.1155/2014/840492

**Published:** 2014-11-19

**Authors:** Manal Mustafa, Nabil Moghrabi, Bassam Bin-Abbas

**Affiliations:** ^1^Department of Pediatrics, Latifa Hospital, Dubai, UAE; ^2^Molecular Diagnostic Laboratory, King Faisal Specialist Hospital and Research Center, Riyadh 11211, Saudi Arabia; ^3^Department of Pediatrics, King Faisal Specialist Hospital and Research Center, MBC 58, P.O. Box 3354, Riyadh 11211, Saudi Arabia

## Abstract

FGFR3 mutations cause wide spectrum of disorders ranging from skeletal dysplasias (hypochondroplasia, achondroplasia, and thanatophoric dysplasia), benign skin tumors (epidermal nevi, seborrhaeic keratosis, and acanthosis nigricans), and epithelial malignancies (multiple myeloma and prostate and bladder carcinoma). Hypochondroplasia is the most common type of short-limb dwarfism in children resulting from fibroblast growth factor receptor 3 (FGFR3) mutation. Acanthosis nigricans might be seen in severe skeletal dysplasia, including thanatophoric dysplasia and SADDAN syndrome, without a biochemical evidence of hyperinsulinemia. Insulin insensitivity and acanthosis nigricans are uncommonly seen in hypochondroplasia patients with FGFR3 mutations which may represent a new association. We aim to describe the association of hypochondroplasia, acanthosis nigricans, and insulin resistance in a child harboring FGFR3 mutation. To our knowledge, this is the first case report associating the p.N540 with acanthosis nigricans and the second to describe hyperinsulinemia in hypochondroplasia. This finding demonstrates the possible coexistence of insulin insensitivity and acanthosis nigricans in hypochondroplasia patients.

## 1. Introduction

Fibroblast growth factor receptor 3 (FGFR3) gene germline mutations are well-known causes of skeletal dysplasia syndromes which encompass a wide spectrum of disorders that range from the relatively mild short-limb dwarfism, hypochondroplasia (HCH), to the most common genetic form of dwarfism, achondroplasia (ACH), to severe achondroplasia with acanthosis nigricans and mental retardation (SADDAN syndrome) [1, 2].

FGFR3 encodes a transmembrane tyrosine-protein kinase receptor that acts as cell-surface receptor for fibroblast growth factors. It has an essential role in the regulation of chondrocyte differentiation, proliferation, and apoptosis and is required for the normal skeletal development [3]. Activating FGFR3 mutations will exacerbate the growth-inhibitory action on chondrocytes, resulting in varying degrees of severity of skeletal dysplasia as per mutation type. FGFR3 germline mutations cause skeletal disorders, while its somatic mutations have been identified in benign skin lesions and epithelial neoplasms. The mutagenic effect of FGFR3 receptors may result in various skin disorders and cancer including epidermal nevi, seborrheic keratosis, and acanthosis nigricans (AN) [1, 2]. Cell-specific pathways may modulate the effect of activating FGFR3 mutations in each cell type.

Acanthosis nigricans is associated with severe skeletal dysplasia caused by activating germline mutations of FGFR3 gene, including thanatophoric dysplasia and SADDAN syndrome [1]. Insulin insensitivity with secondary hyperinsulinemia is uncommonly seen in HCH patients with FGFR3 mutations which may represent a new association.

## 2. Case Presentation

Our patient was a boy who was referred to pediatric endocrinology clinic at the age of three years and 10 months for evaluation of short stature. He was born at term to first degree consanguineous healthy parents. Pregnancy was uncomplicated and the delivery was by cesarean section. His birth weight was 3.7 kg (SDS +0.7), birth length was 50 cm (SDS +0.1), and head circumference was 35 cm (SDS +0.4). He had an elder healthy male sibling with normal stature. His midparental height was 167 cm.

He was otherwise normal and he had attained normal developmental milestones. His growth was uneventful until 3 years of age, when it slowed substantially. At the age of 3 years and 10 months, his weight was 15 kg (SDS −0.5), height was 86 cm (SDS −3.7), and growth velocity was 3 cm/year. He had subtle facial dysmorphic features, disproportionate short stature (upper to lower segment ratio 1.5), and short upper limbs (rhizomelia). He was prepubertal and his systemic examination was unremarkable.

**Figure 1:**
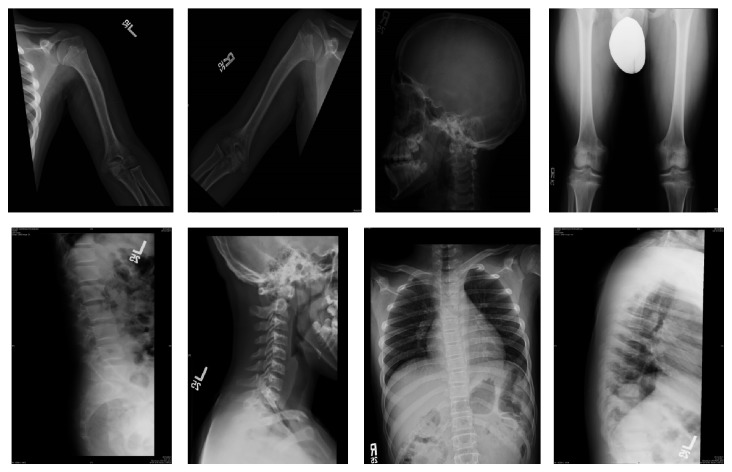
Patient's skeletal survey in favor of hypochondroplasia.

He had normal renal and hepatic function tests and negative celiac antibody screening. His insulin growth factor-1 (IGF-1) and IGF binding protein-3 (IGFBP-3) were normal for age. Growth hormone (GH) levels on insulin and clonidine provocation tests were normal. His hormonal assessment showed normal thyroid stimulating hormone (TSH), free thyroxine (T4), adrenocorticotrophic hormone (ACTH), and cortisol. Bone age was appropriate for chronological age. The radiological study of the skeleton was concordant with the diagnosis of hypochondroplasia. It showed mild thickening of skull calvarium, mild midfacial hypoplasia, rhizomelic shortening of humeri, femurs, and tibias bilaterally. It also demonstrated normal bilateral radii, ulnae, hands and feet. The spinal X-ray showed short pedicles with mild posterior scalloping of the upper lumbar vertebrae and lack of interpedicular distances on the AP view of the lumbar spine (Figure 1).

**Figure 2:**
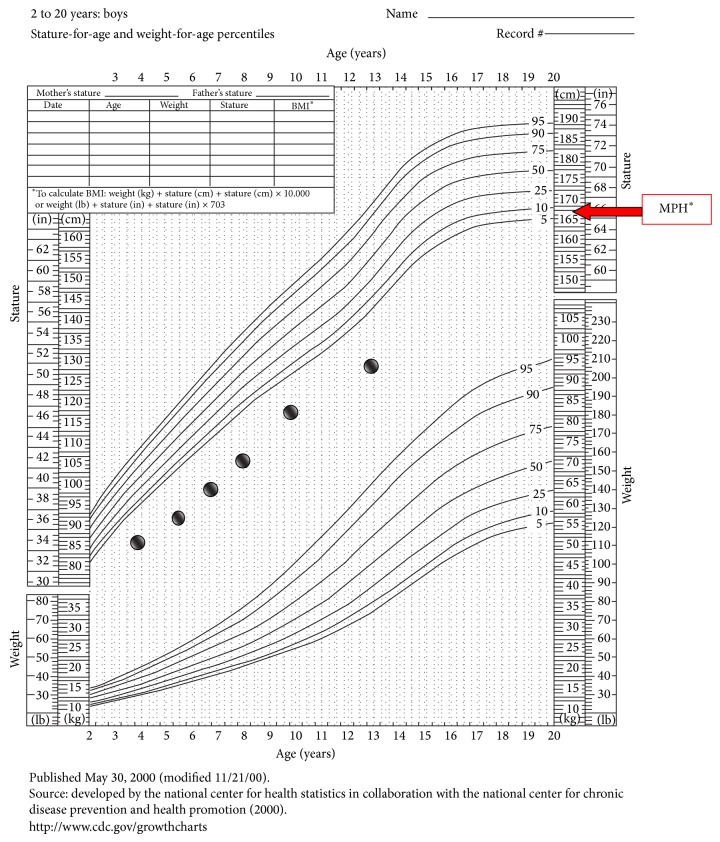
Patient's growth chart.  ^*^Midparental height.

Based on his initial clinical assessment and investigations, he was diagnosed with hypochondroplasia. During his follow-up clinic visits, he was noticed to have suboptimal growth velocity, and his height deviated from his genetic height potential. At the age of seven years, his height was 98 cm (−4.6 SDS) and his growth velocity was 3 cm/year. In view of his short stature, persistent low growth velocity, he was started on recombinant human growth hormone treatment with a dose of 0.03 mg/kg/day which was later adjusted to 0.05 mg/kg/day during follow-up clinic visits. His growth velocity improved to 5 cm per year; however, his height remained below SDS −3.0 (Figure 2).

**Figure 3:**
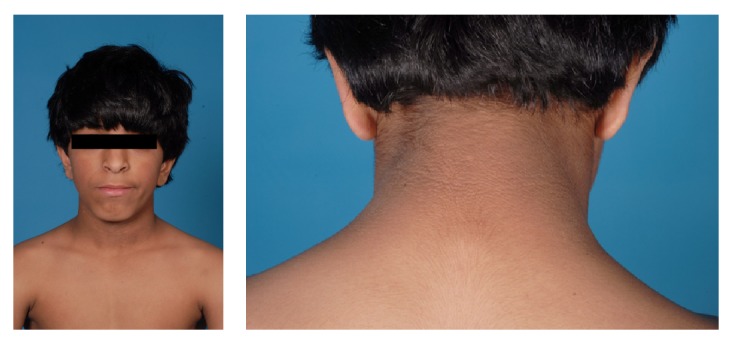
Acanthosis nigricans around the patient's neck.

At the age of 9 years and 10 months, after almost 3 years of growth hormone treatment, he developed normoinsulinemic acanthosis nigricans around his neck (Figure 3). His weight was 31.1 kg (50th percentile), height was 117.2 cm (−3.3 SDS), and body mass index (BMI) was 22.6 kg/m^2^ (+1.6 SDS). A 1.75 g/kg oral glucose tolerance test (OGTT) showed normal serum glucose, fasting and two hours after ingestion of a glucose load. His glycosylated hemoglobin (HBA1C) was normal 5.6% with normal fasting serum insulin 43.2 pmol/L (normal range 17.8–173 pmol/L).

**Figure 4:**
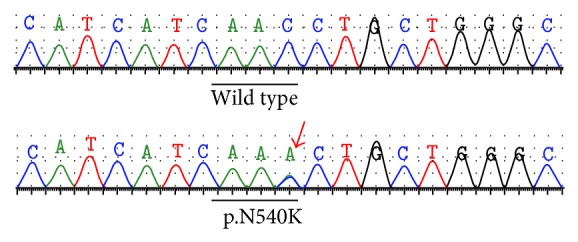
Electropherogram showing the heterozygous base change from cytosine to adenine (arrow) in the FGFR3 gene, which leads to the substitution of asparagine (N) to lysine (K) at codon 540 of the FGFR3 protein.

At the age of 13 years, his anthropometric measurements were as follows: weight 45.6 kg (50th percentile), height 130.5 cm (SDS −3.3), and BMI 26.8 kg/m^2^ (+1.9 SDS). His investigations were repeated, HBA1C was 5.5%, fasting blood glucose 5.2 mmol/L, but his fasting serum insulin was higher 111.0 pmol/L (15.8 *μ*U/L). The homeostasis assessment index for insulin resistance (HOMA-IR) was 3.69 (normal range 0.36–2.41) indicating insulin resistance. His bone age was corresponding to 13.0 years. FGFR3 gene mutation was suspected. Genomic DNA was extracted from peripheral blood leucocytes (Qiagen Inc., Chatsworth, CA), followed by PCR and direct sequencing. A heterozygous c.1620C>A transversion was identified resulting in asparagine to lysine (p.N540K) substitution in tyrosine kinase domain of the FGFR3 (Figure 4). Testing of parents DNA was negative suggesting a de novo mutation.

## 3. Discussion

Hypochondroplasia (HCH; MIM# 146000) is a mild form of skeletal dysplasia caused by heterozygous activating germline mutations in FGFR3 [1]. It is inherited in an autosomal dominant manner, and in the majority of new cases of HCH, the mutations appear de novo.

Fibroblast growth factor receptor 3 (FGFR3) is a member of a family that comprises four related receptors (FGFR1-4). FGFR3 mutations may lead to a wide spectrum of disorders ranging from skeletal dysplasias (hypochondroplasia, achondroplasia, and thanatophoric dysplasia), benign skin tumors (epidermal nevi, seborrheic keratosis, and acanthosis nigricans), and epithelial malignancies (multiple myeloma, prostate and bladder carcinoma, and seminoma) [2]. FGFR3 germline mutations cause autosomal dominant skeletal disorders, while its somatic mutations have been identified in benign skin lesions and epithelial neoplasms. Germline mutations of FGFR3 cause severe skeletal dysplasia syndromes. R248C, S249C, G372C, S373C, and K652E germline mutations are linked to thanatophoric dysplasia, and affected individuals usually die as neonates. Two other germ line* FGFR3* mutations cause Crouzon (A393E) and severe achondroplasia with developmental delay and acanthosis nigricans (K652M) “SADDAN syndrome.” In both syndromes, the patients develop normoinsulinemic acanthosis nigricans [3]. By contrast, p.Lys650Asn and p.Lys650Gln mutations that activate the FGFR3 to a lesser degree are associated with milder forms of skeletal dysplasia, such as hypochondroplasia.

FGFR3 is a physiological negative regulator of skeletal growth, which restricts the length of long bones via inhibition of chondrocyte proliferation. Based on that, the profound dwarfing phenotypes in individuals carrying gain-of-function mutations in FGFR3 are not due to novel FGFR3 functions in cartilage but rather resemble an exaggeration of its physiological roles. The occurrence of somatic FGFR3 mutations in benign skin tumors and epithelial malignancies is caused by excessive cell proliferation and suggests a promitogenic FGFR3 effect on keratinocytes, melanocytes, and epithelial cells. Because the growth-inhibitory action of FGF signaling is specific to chondrocytes, whereas mitogenic for other cell types such as cells in the bladder epithelium or keratinocytes, this explains the fact that only severe forms of skeletal dysplasias had been associated with AN.

FGFR3 inhibits the expansion of the immature pancreatic epithelium in murine pancreatic development, whereas in keratinocytes the increased kinase activity results in expression of antiapoptotic proteins and expansion of the epidermal component. Altogether, the growth-inhibitory role of FGFR3 in cartilage appears unique when compared to its effects in other tissues such as cells of mesenchymal origin. Cell-specific pathways may modulate the effect of activating FGFR3 mutations in each cell type.

Acanthosis nigricans is a velvety and papillomatous pigmented hyperkeratosis of the skin, which appears mainly on the flexures and neck. It is commonly observed in conditions associated with reduced insulin sensitivity such as obesity, lipodystrophy, and acromegaly [4, 5] and it is a criterion for identifying children at risk of type 2 diabetes mellitus [6]. It results from the stimulation of keratinocytes and skin fibroblasts by the activation of growth factor receptors, such as epidermal growth factors, insulin-like growth factors (IGF1), and fibroblast growth factors [7]. Somatic FGFR3 mutations have been detected in 40% of seborrheic keratoses (SKs) of the hyperkeratotic and acanthotic subtypes, which are very common benign skin tumors. The mechanism for the high rate of somatic FGFR3 mutations in these benign skin tumors remains elusive, but UV light exposure may play a potential role.

Growth hormone treatment can also induce AN by stimulating the production of IGF1. Activation of IGF1 receptors can lead to the growth and differentiation of many different cell lines, including keratinocytes causing AN development [8].

Previous case reports described the association of normoinsulinemic acanthosis nigricans with achondroplasia and hypochondroplasia [9–13]. One of the early reports described a mutation in codon 650 of FGFR3 in a patient with mild form of osteochondrodysplasia and AN (p.K650Q) [9]. Berk et al. described four relatives who had familial AN without skeletal abnormalities and were found to have FGFR3 mutation (p.K650T) [10]. Castro-Feijóo et al. reported ten affected family members with HCH and AN due to p.K650T mutation. Four of them aged 16–46 years had normal oral glucose tolerance, while the other five had normal fasting glucose and insulin level and one patient was diagnosed with adult onset diabetes mellitus [11]. Their finding demonstrates the coexistence of both conditions due to the same mutation which might represent a true complex.

There were isolated case reports of ACH with AN developed either during treatment with growth hormone or without previous history of treatment [12, 13]. In a recent case series of five male patients with AN in ACH and HCH due to FGFR3 mutations, it was found that all of them had normal insulin sensitivity compared with puberty-matched controls [14]. There was a single case report of a ten-year-old boy with ACH who developed AN during treatment with GH [12]. On the other hand, another study of 35 children with ACH who received GH for five years did not show any evidence of increased insulin resistance or development of AN during the study period [15]. A single case report by Blomberg et al. described a 14-year-old girl with mild hypochondroplasia due to K650Q mutation in the FGFR3 gene, who developed acanthosis nigricans and hyperinsulinemia [16]. He concluded that FGFR3 mutation analysis should be considered in case of the coexistence of acanthosis nigricans and a skeletal dysplasia and advised testing for hyperinsulinemia, especially if FGFR3 gene mutation is confirmed.

Different mutations in FGFR3 have been identified in HCH patients. p.N540K substitution in the FGFR3 gene accounts for about 70% of HCH cases. Three other missense mutations affecting the same p.N540 codon were also reported in patients with HCH; c.1620C>G (p.N540K) [17]; c.1619A>G (p.N540S) [18]; and c.1619A>C (p.N540T) [19]. To the best of our knowledge, this is the first report associating the p.N540 with AN and the second to describe hyperinsulinemia in hypochondroplasia.

All previous studies could not conclude whether the development of AN in ACH/HCH is due to reduced insulin sensitivity secondary to the skeletal dysplasia or GH treatment. Given the complexity of FGFR3 downstream signaling, the mechanism involved in the development of AN in these patients is still unclear.

## 4. Conclusion

Given the complexity of FGFR3 downstream signaling, the mechanism involved in the development of AN in HCH patients is still unclear. Our findings suggest that it might be due to insulin insensitivity either related to skeletal dysplasia itself or secondary to treatment with recombinant human GH or may represent a new association that should be established by further studies. Hyperinsulinism found in our patient could be merely a possible chance; however, testing for hyperinsulinemia in hypochondroplasia patients is otherwise advised. Long-term follow-up for HCH patients is needed to clarify the risk of long-term metabolic complications.
